# Measuring microRNA-371a-3p in testicular germ cell tumors–making the test ready for clinical routine practice

**DOI:** 10.1007/s12672-026-05345-x

**Published:** 2026-06-02

**Authors:** Axel Heidenreich, Tabea Niemzok, Martin Kämper, Gazanfer Belge, Markus Klemke, Klaus-Peter Dieckmann, Jan Diesend

**Affiliations:** 1 Department of Urology, Uro-Oncology, Robot-Assisted and Specialized Urologic Surgery, University Hospital of Cologne, Cologne, Germany; 2 Research & Development, mir|detect GmbH, Bremerhaven, Germany; 3 Research & Development, Sarstedt AG & Co. KG, Nümbrecht, Germany; 4 Department of Tumor Genetics, Faculty of Biology and Chemistry, University of Bremen, Bremen, Germany; 5 Department of Urology, Asklepios Klinik Altona, Hamburg, Germany

**Keywords:** miR-371a-3p, Testicular germ cell tumor, Liquid biopsy, Preanalytical handling

## Abstract

The M371-Test enables standardized measurement of miR-371a-3p in serum, which represents a highly sensitive and specific biomarker for testicular germ cell tumors (GCTs). The current protocol requires immediate centrifugation, freezing, and cold-chain shipping to the laboratory, which poses significant barriers to practical use of the test. Previous studies suggested that plasma from the S-Monovette cfDNA Exact may offer a simplified preanalytical pathway with extended sample stability at ambient temperature, potentially improving test accessibility. To evaluate this approach, we are conducting a prospective multicenter study with a target enrollment of ≥ 250 patients comparing M371-Test performance in paired plasma and serum samples; this report presents an interim analysis of 109 eligible patients (67 GCT cases, 42 controls). M371-Test performance in plasma was very similar to serum, with sensitivities of 92.5% and 94.0%, respectively, while specificity was 100% in plasma versus 92.9% in serum. Subgroup analysis showed that the difference in specificity was concentrated in the post-treatment remission subgroup (84.6% serum vs. 100% plasma). These findings indicate that plasma and serum yield comparable diagnostic performance with the M371-Test. The simplified preanalytical workflow associated with the S-Monovette cfDNA Exact warrants formal stability and robustness evaluation in the completed study. If confirmed in the full cohort, this approach may offer a more practical pathway for miR-371a-3p-based GCT diagnostics in routine practice.

**Supplementary Information:**

The online version contains supplementary material available at 10.1007/s12672-026-05345-x.

Over the past decade, miR-371a-3p has emerged as a highly promising biomarker for germ cell tumor (GCT) diagnosis and surveillance, substantially outperforming α-fetoprotein (AFP), β-human chorionic gonadotropin (β-hCG), and lactate dehydrogenase (LDH) [1]. The M371-Test is a CE-marked assay available since 2020 and IVDR-compliant since 2024, and it enables standardized clinical testing of miR-371a-3p with 94% sensitivity and 96% specificity for initial diagnosis [2], improving to 100% sensitivity and 96% specificity for monitoring [3], and objective identification of marker-negative, small-volume retroperitoneal metastases [4]. Despite this excellent performance, broad adoption of miR-371a-3p measurements remains constrained by its limitation to serum, which imposes stringent handling requirements and leaves little margin for error [5].

Current preanalytical practice for miR-371a-3p measurements entails 30–60 min of upright incubation, rapid centrifugation, immediate transfer of serum, and freezing to prevent miRNA degradation, followed by frozen shipping (Fig. 1). Delayed processing can induce hemolysis, which releases the reference-miR of the M371-Test from erythrocytes potentially skewing results [6]. For many points of care, these requirements present substantial barriers: centrifuges may be unavailable, freezer capacity limited, and frozen sample logistics unfamiliar. Samples must be shipped within 90 h at temperatures not exceeding 0 °C, introducing significant risk from sample handling errors or shipping delays. Above all, manpower and time for processing blood samples in that way are not available in clinical routine.

**Fig. 1:**
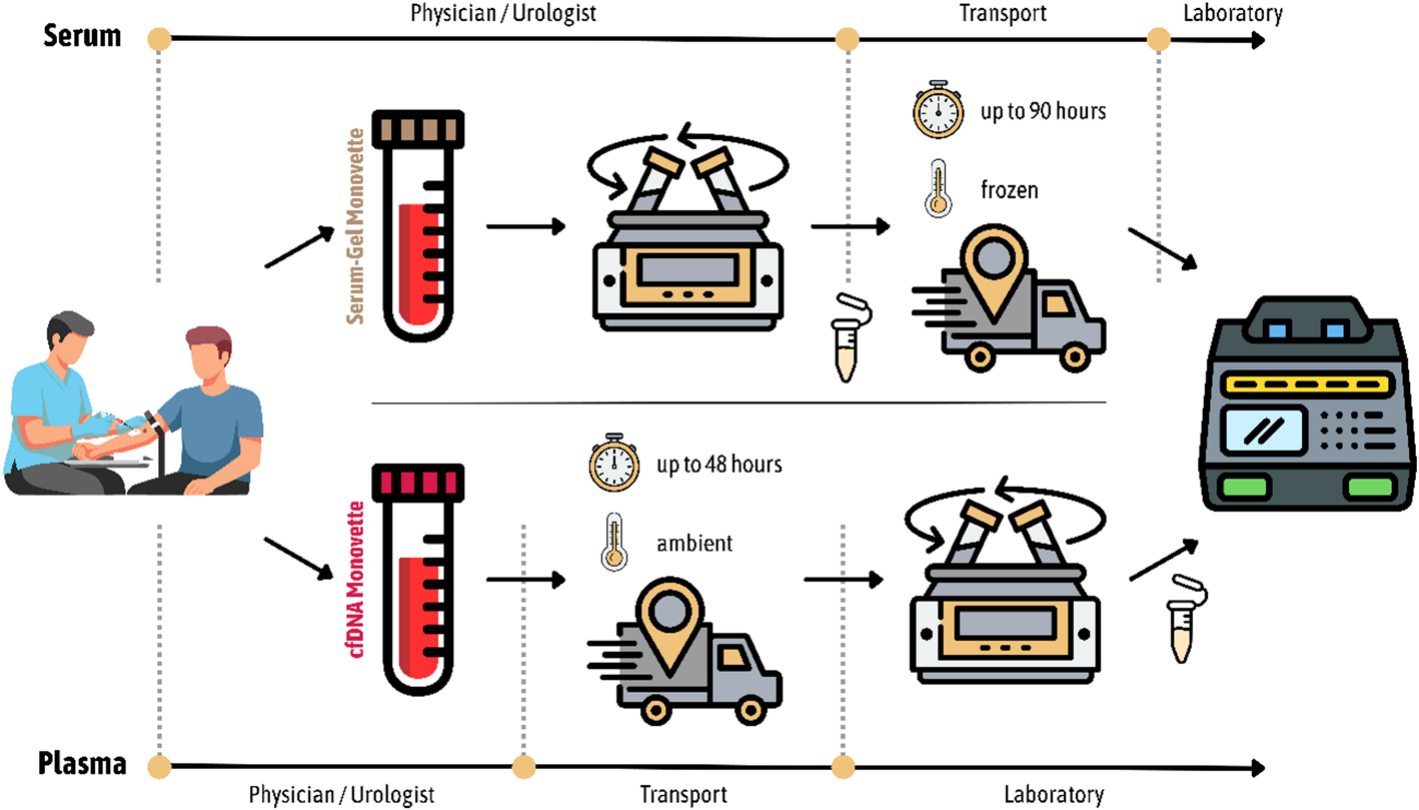
Comparison of preanalytical workflows for serum- and plasma-based miR-371a-3p testing. The serum pathway requires onsite centrifugation within 30–60 min of collection, followed by frozen transport at ≤ 0 °C (or on dry ice), posing significant logistical challenges in outpatient settings. By contrast, the plasma pathway permits transport of whole blood, with centrifugation performed at the diagnostic laboratory. This approach allows physicians to utilize established sample transport infrastructure, substantially reducing barriers to test adoption in outpatient care

Initial experience indicated that the use of plasma as matrix for the M371-Test could alleviate most of these preanalytical challenges. In experiments by the manufacturer, stabilized whole blood collected in the S-Monovette cfDNA Exact (Sarstedt, Nümbrecht, Germany) reliably stabilized miRNA for extended periods at elevated temperatures. In practical terms, this experience would suggest shipping whole blood in the S-Monovette cfDNA Exact at ambient temperature immediately after collection without centrifugation. This pathway would reduce the preanalytical burden thereby increasing test accessibility, particularly in outpatient settings (Fig. 1).

Beyond improved stability, the plasma pathway offers several additional practical advantages for miR-371a-3p measurements. Critical preanalytical steps would shift from heterogeneous office environments to standardized diagnostic laboratories, enabling integration with established logistical systems. Specialized shipping containers and express transport services would no longer be required, failed analytical runs would decrease through streamlined preanalytics, and staff training requirements at office level would be reduced. Ultimately, sample quality upon laboratory arrival would be more consistent, benefiting both clinicians and patients.

Circulating miRNAs have been reported as highly stable in both serum and plasma under varying environmental conditions [7], and several studies have specifically investigated plasma-based miR-371a-3p measurement [8–10]. While the reported data indicated very similar measurement results in serum and plasma, these investigations employed non-standardized protocols, precluding direct comparison with the M371-Test.

To address this gap, we initiated a prospective multicenter clinical study with a target enrollment of ≥ 250 patients. In this interim analysis, data from 109 eligible patients (67 GCT, 42 controls) indicate that the M371-Test performance in plasma is comparable to that observed in serum. The control cohort (*n* = 42) comprised three predefined subgroups to represent the use in clinical routine: benign testicular conditions (*n* = 17), non-GCT testicular pathologies (*n* = 12), and patients in clinical remission after GCT treatment (*n* = 13). C_T_ values for miR-371a-3p and the reference-miR demonstrated comparable distributions in plasma and serum across GCT patients and the control cohort (Suppl. Figure 2 A, B). Accordingly, calculated RQ values showed strong concordance between matrices, spanning several orders of magnitude in GCT cases while remaining consistently low in the control cohort (Suppl. Figure 2 C). A notable difference emerged in the remission and benign control subgroups: three serum samples yielded borderline positive RQ values (10.27, 17.03, and 18.42), whereas all plasma samples from these patients remained below the diagnostic cut-off of RQ 10.

This observation is reflected in the performance characteristics of the two matrices calculated at the cutoff of RQ = 10 prespecified by the manufacturer. The M371-Test demonstrated equivalent diagnostic performance in plasma and serum (Table 1, Suppl. Figure 3). In serum, sensitivity for detecting all GCTs (*n* = 67) was 94.0% with a specificity of 92.9%. Plasma-based measurements yielded a sensitivity of 92.5% and a specificity of 100%. Subgroup analysis revealed consistent performance across histological subtypes: sensitivity for seminoma (*n* = 39) was 92.3% and 89.7% in serum and plasma, respectively, while sensitivity for nonseminoma (*n* = 28) remained identical at 96.4% in both matrices. This difference between subtypes is consistent with the previously reported lower expression of miR-371a-3p in seminoma compared to nonseminoma, especially in CSI [2]. To address the heterogeneity of the control cohort, performance was further stratified into three predefined subgroups: benign testicular conditions, post-treatment GCT remission, and other non-GCT testicular pathologies (Suppl. Table 2). Subgroup-specific ROC analysis (Suppl. Figure 4, Suppl. Table 2) showed that the three serum false-positive results were distributed across the remission and benign subgroups, while plasma yielded no false-positive results in any control subgroup. The effect was most pronounced in the post-treatment remission subgroup, where serum specificity was 84.6% compared to 100.0% in plasma. False-positive results in this setting carry the greatest clinical implications.

**Table 1:**
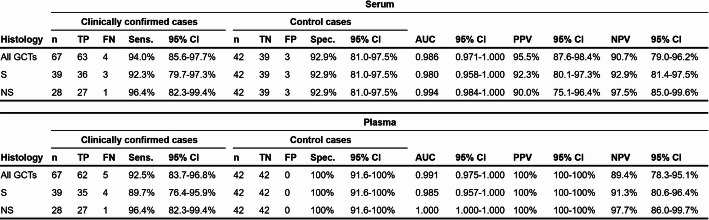
Performance of the M371-Test in plasma and serum

TP: true positive; FN: false negative; TN: true negative; FP: false positive; Sens.: sensitivity; Spec.: specificity; PPV: positive predictive value; NPV: negative predictive value; CI: confidence interval; S: seminoma; NS: nonseminoma

All performance estimates above were calculated using the manufacturer-specified RQ = 10 cutoff applied unchanged to plasma. To assess whether the inherited cutoff was appropriate for plasma, an exploratory ROC-derived optimal cutoff was calculated using the Youden index. The plasma-optimal cutoff was RQ = 2.94, at which sensitivity rose to 97.0% while specificity remained 100.0% in this interim cohort. Given the small number of control cases at this sample size and the established RQ = 10 cutoff for the M371-Test, performance comparisons in this report use the inherited cutoff.

Predictive value analysis further supported these findings (Table 1). Based on a prior probability of 61.5%, the positive predictive value (PPV) was 95.5% in serum compared to 100% in plasma, while negative predictive values (NPV) were comparable at 90.7% and 89.4%, respectively. The 1.5% point difference in sensitivity (92.5% in plasma vs. 94.0% in serum) corresponds to a one- to two-patient difference in this interim cohort and is within the margin expected from sampling variation. Combined with the higher specificity in plasma, the resulting predictive values were comparable to or favorable over those obtained in serum, particularly for nonseminoma where NPVs reached 97.5% (serum) and 97.7% (plasma).

This study has several limitations. First, the comparison was designed at the level of the proposed clinical workflow, with serum collected in standard S-Monovette Serum Gel tubes and plasma collected in S-Monovette cfDNA Exact tubes. The relative contributions of matrix (serum vs. plasma) and collection tube chemistry to the observed performance therefore cannot be separated from this dataset and were not an objective of the present analysis. Second, the control cohort was heterogeneous, including benign testicular conditions, post-treatment GCT remission, and non-GCT testicular pathologies; subgroup numbers limit interpretation of subgroup-specific specificity. Third, the RQ = 10 cutoff applied to plasma was inherited from the validated serum cutoff and was not independently validated in plasma; an exploratory plasma-derived optimal cutoff is reported alongside the fixed cutoff for transparency and will be reassessed in the full study cohort. Fourth, predictive values were calculated using the disease prevalence within the study cohort (61.5%); applicability to lower-prevalence clinical settings (e.g., post-treatment surveillance) requires separate evaluation. Confirmation in the full prespecified cohort is ongoing.

In summary, these preliminary findings demonstrate that M371-Test performance in plasma is comparable to that in serum, with equivalent sensitivity and specificity. The absence of false-positive results in plasma, combined with the simplified preanalytical requirements of the S-Monovette cfDNA Exact, supports the feasibility of plasma-based M371-Test application. The simplified preanalytical workflow associated with the cfDNA Exact tube avoids the stringent temperature control and rapid processing required for serum to preserve miRNA integrity. Formal stability evaluation will be reported in the full study. These advantages are particularly attractive for decentralized healthcare settings and resource-limited environments where adherence to strict preanalytical protocols remains challenging. Upon completion of the target enrollment of ≥ 250 patients, the complete dataset will provide the basis for the prespecified primary analysis of plasma-based M371-Test application. The observed performance reflects the combined effect of plasma matrix and the cfDNA Exact stabilizer. We report these interim findings to inform about ongoing method development; this approach may offer a more accessible pathway for miR-371a-3p testing in patients with GCTs.

## Supplementary Information

Below is the link to the electronic supplementary material.

Supplementary Material 1.

## Data Availability

The data that support the findings of this study are not openly available due to reasons of sensitivity and are available from the corresponding author upon reasonable request.
