# Supplementary material for: Measuring microRNA-371a-3p in testicular germ cell tumors–making the test ready for clinical routine practice
Source: Discov Oncol. 2026 Jun 2;17:840. doi: 10.1007/s12672-026-05345-x (PMC13230357; doi:10.1007/s12672-026-05345-x)
Supplement: Supplementary file 1 — Supplementary Material 1. [file 12672_2026_5345_MOESM1_ESM.docx]

**MATERIALS AND METHODS**

**Study design and participants**

Between July 2023 and December 2025, 147 patients with GCT and 42 patients within the control cohort were prospectively enrolled from two urologic institutions in Germany (University Hospital Cologne and Asklepios Klinik Altona). Of those, 80 patients were subsequently excluded, yielding a final study cohort of 109 patients. Exclusion criteria were missing clinical information, missing samples, multiple samples of one patient, and patients with teratoma. The final GCT population (n=67) consisted of 39 patients with seminoma and 28 patients with non-seminoma. The control cohort (n=42) comprised three predefined subgroups: benign testicular conditions (n=17), non-GCT testicular pathologies (n=12), and patients in clinical remission after GCT treatment (n=13). The study population is visualized in Supplemental Figure 1. The baseline characteristics of the final population are described in Supplemental Table 1. Caregivers of study patients were blinded to measurement results of the M371-Test, thus no clinical decision-making was based on study results.

Ethical approval was provided by the Ethical committee of Ärztekammer Hamburg (MC 152/19, July 15, 2019) and by Ärztekammer Bremen (#301/17, September 21, 2017). Written informed consent was obtained from all patients. All study activities were conducted in accordance with the Declaration of Helsinki of the World Medical Association, amended by the 64^th^ General Assembly in October 2013.

**Sample collection**

Paired blood samples were collected from each patient: serum in 9.0 mL S-Monovette® Serum Gel tubes (Sarstedt, Nümbrecht, Germany) and plasma in 9.2 mL S-Monovette® cfDNA Exact tubes (Sarstedt). The S-Monovette cfDNA Exact tube was selected based on prior internal and Sarstedt feasibility data demonstrating the consistent stabilization of miR-371a-3p and the reference-miR among the available tube formats at study initiation. Both tubes were used according to the manufacturer’s instructions. For serum preparation, samples were allowed to clot for 30 minutes at room temperature prior to centrifugation at 2,000 × g for 10 minutes at 20°C. Transport of serum was conducted in a frozen state. Plasma was isolated through a two-step centrifugation protocol: an initial centrifugation at 2,500 × g for 10 minutes following replacement of the tube cap, and a second centrifugation at 15,000 × g for 15 minutes to ensure platelet depletion. Notably, plasma samples were maintained at ambient temperature throughout processing and transport, as preliminary observations had indicated that refrigerated storage of S-Monovette® cfDNA Exact tubes prior to centrifugation may induce hemolysis, potentially compromising reference miRNA measurements. Both serum and plasma aliquots were stored at −80°C until analysis.

**miR-371a-3p measurement**

The miRNA fraction was isolated from 200 µL of cubital vein serum or plasma using the miRNeasy Serum/Plasma Kit (Qiagen, Hilden, Germany) according to the manufacturer's instructions. Quantification of miR-371a-3p and the endogenous reference-miR was performed using the M371-Test (mir|detect, Bremerhaven, Germany) on a LightCycler PRO instrument (Roche Diagnostics, Mannheim, Germany). The relative quantity (RQ) of miR-371a-3p was calculated using the ΔΔCt method in the evaluation software supplied by the manufacturer.

**Statistical analysis**

All analyses were performed in R version 4.5.1 (R Foundation for Statistical Computing, Vienna, Austria). ROC analyses were performed with the pROC package 1.19.0.1, paired comparisons with base R, and figures generated with ggplot2 4.0.3, extended with ggh4x 0.3.1 for nested axis labels and RColorBrewer 1.1-3 for color palettes.

*Diagnostic performance.* Sensitivity and specificity were calculated against the histopathological reference (GCT vs. control). The fixed cutoff of RQ = 10, originally established and validated in serum and specified in the manufacturer's instructions for use, was applied unchanged to plasma to enable direct comparison between matrices.

*ROC analysis.* ROC curves were constructed from RQ values using the pROC package, with empirical (non-parametric) AUC estimation. AUC point estimates and 95% confidence intervals were calculated using the DeLong method. Sensitivity and specificity were derived at the manufacturer-specified cutoff of RQ = 10. To assess whether this inherited cutoff was appropriate for plasma, an exploratory plasma-specific cutoff was derived from the plasma ROC curve using the Youden index. To address heterogeneity within the control cohort, separate ROC analyses were performed for GCT cases versus each control subgroup (benign testicular conditions, post-treatment GCT remission, other non-GCT testicular pathologies), in addition to the analysis against the full control cohort. AUC values between matrices are reported descriptively; formal statistical comparison of paired ROC curves (e.g., DeLong test) was not undertaken at this interim stage.

*Confidence intervals.* 95% confidence intervals were calculated for all reported metrics. Clopper-Pearson (exact binomial) intervals were used for sensitivity, specificity, positive predictive value, and negative predictive value. AUC confidence intervals were computed using the DeLong method.

*Missing data and exclusions.* Within the analyzed cohort, samples with no amplification of miR-371a-3p (Ct undetected) were assigned RQ = 0 and counted as test-negative for diagnostic performance calculations. No imputation was performed. Pairs missing a value in one matrix were retained in all analyses for which both values were available; no imputation was performed.

*Subgroup analyses.* Histological subgroup analyses (seminoma, nonseminoma) and control subgroup analyses (benign, remission, other non-GCT) were prespecified. All subgroup metrics are reported with the same RQ = 10 cutoff and the same CI methods as the overall analysis. Given small per-subgroup sample sizes, subgroup CIs are interpreted descriptively rather than for formal hypothesis testing.

*Paired data structure.* Plasma and serum samples were drawn from each patient at the same timepoint and therefore represent paired observations. Comparison between matrices was performed at the level of diagnostic performance metrics (sensitivity, specificity, AUC) rather than through formal statistical testing of paired Ct or RQ values. Distributional differences between matrices are presented descriptively in Suppl. Fig. 2.


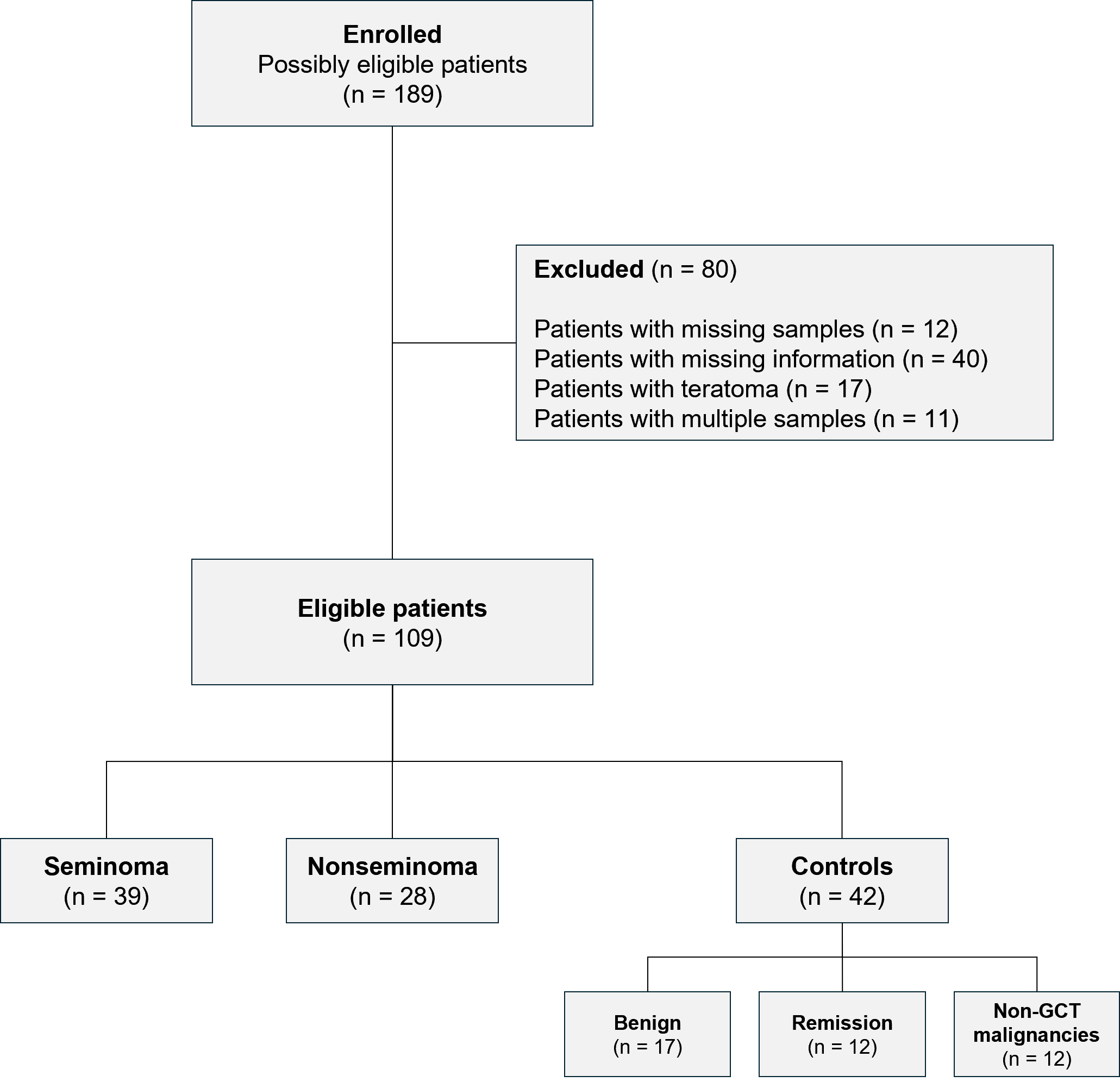


**Supplemental Figure 1. CONSORT diagram for study cohort.**

**Supplemental Table 1. Baseline characteristics of the study cohort.** Cohort composition by histology and control subgroup. Continuous variables are summarised as median (interquartile range, IQR); categorical variables as n (%). Tumor size refers to the maximum diameter of the primary testicular lesion. Clinical stage applies to the GCT cohort only.

| **Characteristic** | **Seminoma (n=39)** | **Nonseminoma (n=28)** | **Benign (n=17)** | **Remission (n=13)** | **Non-GCT pathologies (n=12)** |
| --- | --- | --- | --- | --- | --- |
| **Age, years** |  |  |  |  |  |
| Median (IQR) | 40 (32–56) | 32 (28–40) | 48 (31–70) | 48 (36–56) | 46 (40–62) |
| Range | 25–71 | 19–87 | 20–85 | 21–81 | 20–83 |
| **Clinical stage, n (%)*** |  |  |  |  |  |
| CS1 | 30 (76.9%) | 14 (50.0%) | — | — | — |
| CS2 | 9 (23.1%) | 9 (32.1%) | — | — | — |
| CS3 | 0 (0.0%) | 5 (17.9%) | — | — | — |
| **Tumor size, mm** |  |  |  |  |  |
| Median (IQR), n† | 38 (21–50), 24 | 30 (16–38), 15 | — | — | — |

** Clinical stage according to UICC/AJCC TNM classification. † Tumor size was not consistently recorded for all GCT cases in the source data; n indicates the number of patients with the variable available, and summary statistics are calculated from these only.*


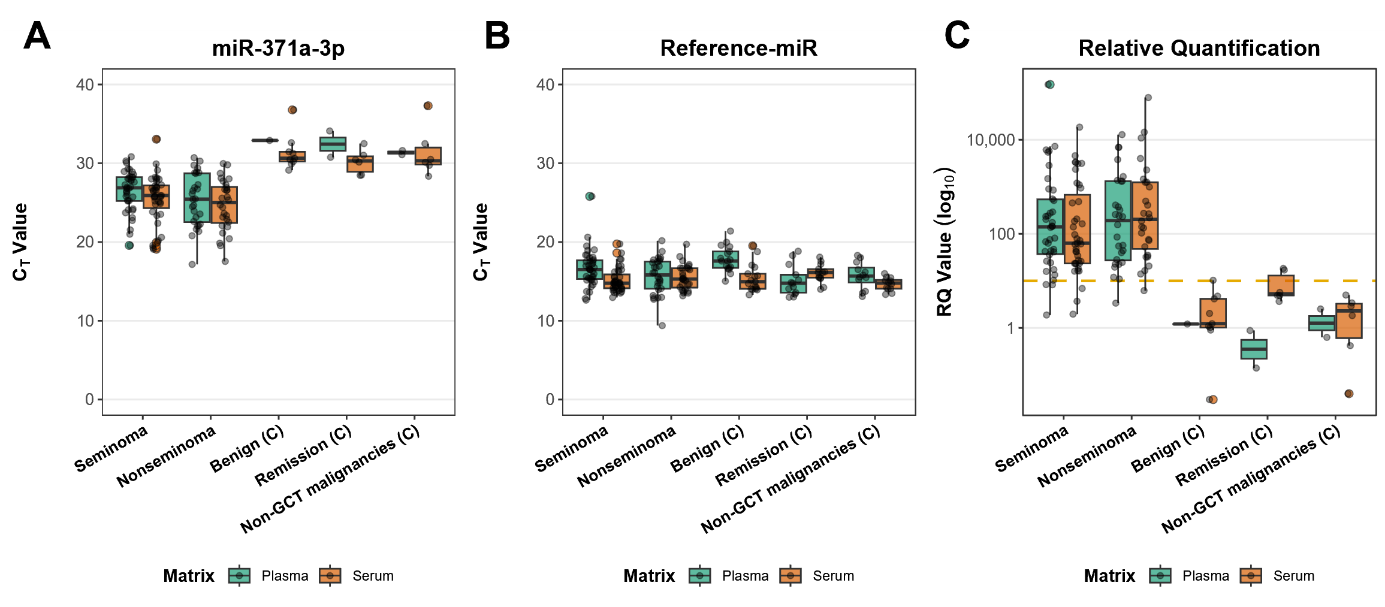


**Supplemental Figure 2. C_T_ values (A-B) and RQ (C) values for miR-371a-3p and the reference-miR in plasma and serum**. The dashed yellow line in **(C)** indicates the cut-off of RQ 10 as specified in the manufacturer's instructions for use. Control groups are split in ‘Benign’, ‘Remission’, and ‘Non-GCT pathologies’ and marked with a C.


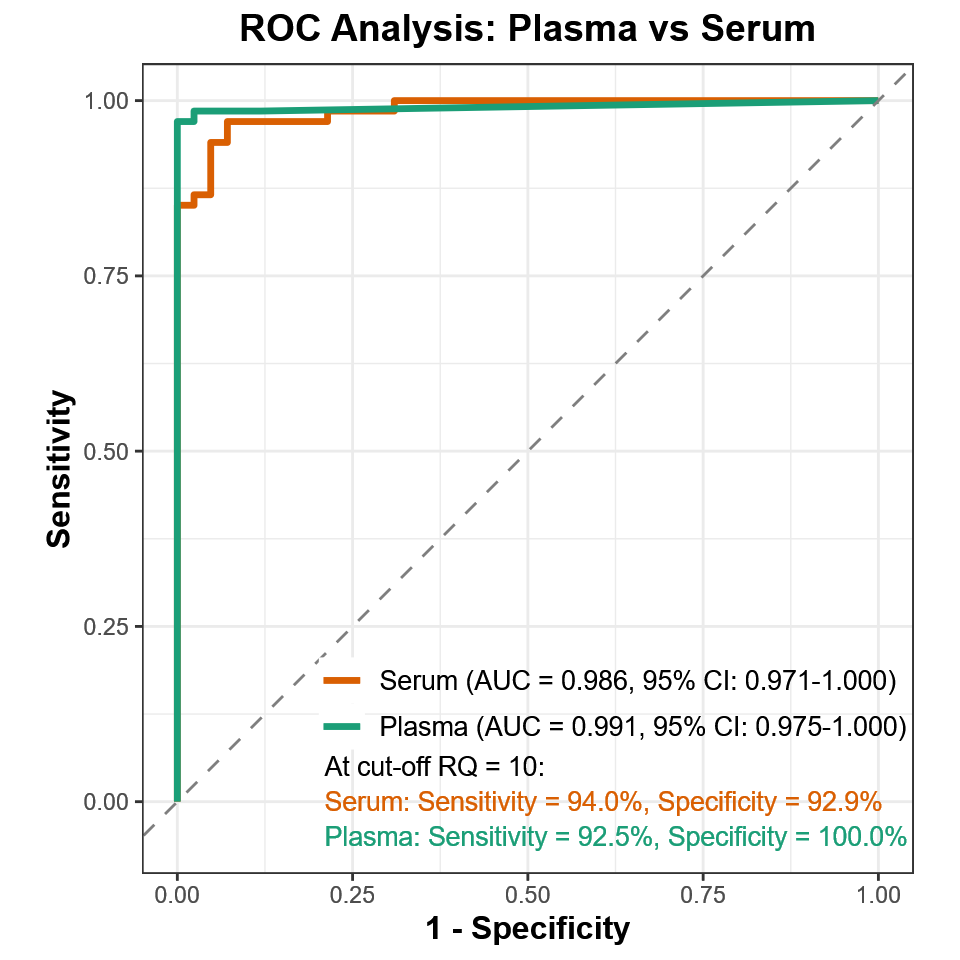


**Supplemental Figure 3. ROC analysis comparing M371-Test performance in plasma and serum**.


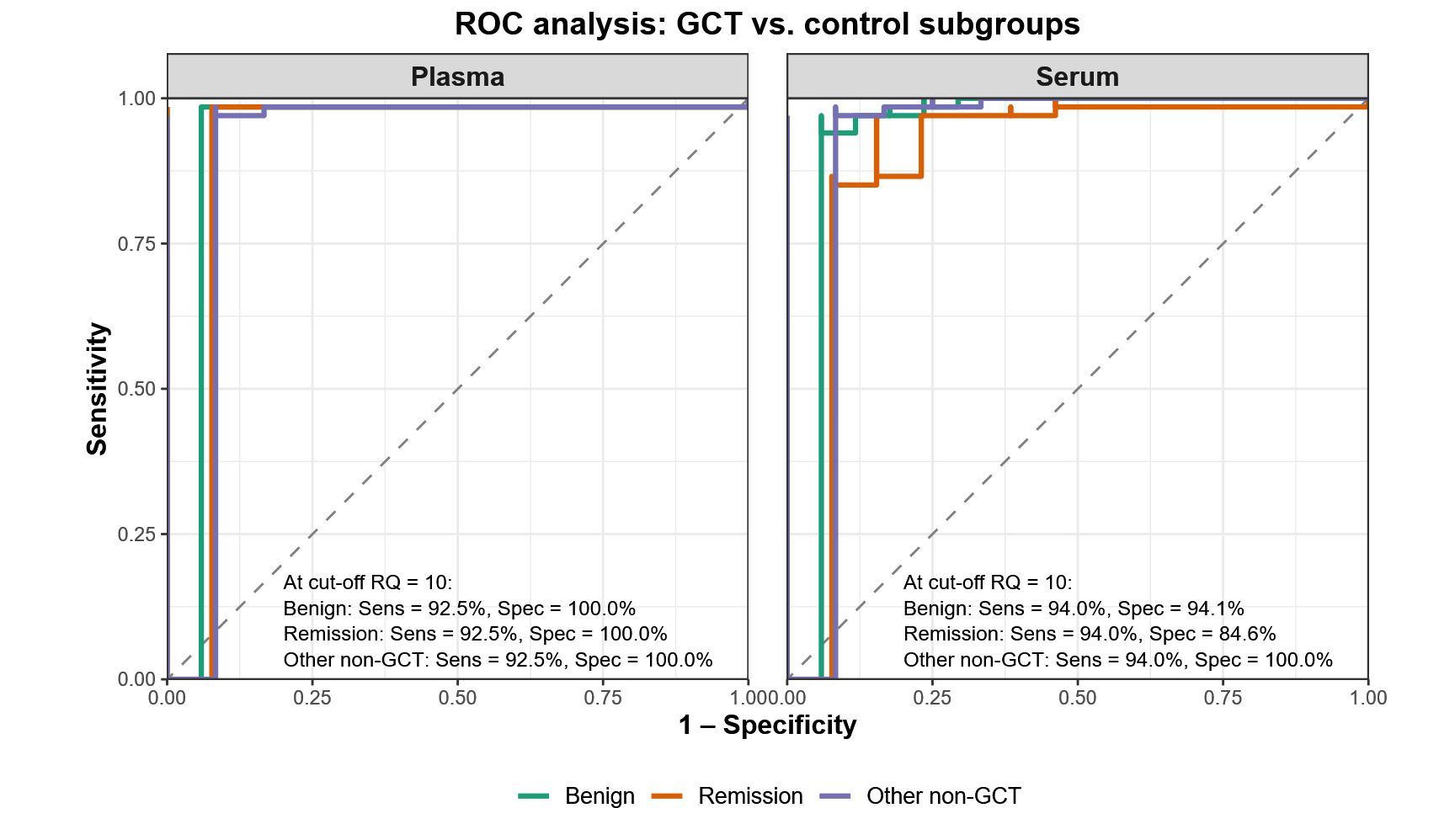


**Supplemental Figure 4. ROC analysis against the control subgroups.**

**Supplemental Table 2. Diagnostic performance of the M371-Test against control subgroups.** Sensitivity, specificity, and AUC are reported for plasma and serum at the manufacturer-specified cutoff of RQ = 10. Control subgroups: benign testicular conditions (n=17), post-treatment GCT remission (n=13), other non-GCT testicular pathologies (n=12; ‘Non-GCT’ in table).

| **Matrix** | **GCT cohort** | **Controls** | **Sensitivity** | **95% CI** | **Specificity** | **95% CI** | **AUC** | **95% CI** |
| --- | --- | --- | --- | --- | --- | --- | --- | --- |
| Serum | All GCTs | Benign | 94.0% | 85.4-98.3% | 94.1% | 71.3-99.9% | 0.992 | 0.980-1.000 |
| Plasma | All GCTs | Benign | 92.5% | 83.4-97.5% | 100.0% | 80.5-100.0% | 0.992 | 0.977-1.000 |
| Serum | All GCTs | Remission | 94.0% | 85.4-98.3% | 84.6% | 54.6-98.1% | 0.970 | 0.936-1.000 |
| Plasma | All GCTs | Remission | 92.5% | 83.4-97.5% | 100.0% | 75.3-100.0% | 0.991 | 0.974-1.000 |
| Serum | All GCTs | Non-GCT | 94.0% | 85.4-98.3% | 100.0% | 73.5-100.0% | 0.995 | 0.986-1.000 |
| Plasma | All GCTs | Non-GCT | 92.5% | 83.4-97.5% | 100.0% | 73.5-100.0% | 0.990 | 0.972-1.000 |
